# Validity of Palmar Pallor for Diagnosis of Anemia among Children Aged 6–59 Months in North India

**DOI:** 10.1155/2014/543860

**Published:** 2014-11-09

**Authors:** Arun Kumar Aggarwal, Jaya Prasad Tripathy, Deepak Sharma, Ajith Prabhu

**Affiliations:** School of Public Health, Postgraduate Institute of Medical Education and Research, Chandigarh 160012, India

## Abstract

*Introduction.* The Integrated Management of Childhood and Neonatal Illness (IMNCI) recommends the use palmar pallor to diagnose anaemia. Earlier studies to validate palmar pallor as clinical sign for anaemia were largely done in African context. There was a need to test validity of palmar pallor to detect anemia in different settings.* Objective.* To study the validity and interobserver agreement of palmar pallor examination to diagnose anemia in children under 5 years of age in India.* Methods. *In a village in Northern India, hemoglobin estimation was done for 80 children using cyanomethemoglobin method. Two examiners, a physician and a health worker, trained in IMNCI evaluated children for palmar pallor. Sensitivity and specificity and Kappa statistics were calculated.* Results.* Health worker diagnosed palmar pallor with sensitivity of 30.8–42.8% and specificity of 70–89%. Similar figures for doctor were 40–47% and 60–66%, respectively. Kappa agreement between a health worker and a physician was 0.48 (95% CI = 0.298–0.666) and then increased to 0.51 when categories of severe pallor and mild pallor were merged.* Conclusion.* While using palmar pallor as clinical sign for anaemia, children with no pallor should also be followed up closely for possible detection of missed cases during follow-up.

## 1. Introduction

Anemia is a major public health problem in India with almost 7 in 10 children aged 6–59 months being anemic [1]. The Integrated Management of Childhood Illness (IMCI) recommends the use of simple clinical sign like palmar pallor to diagnose anemia [2]. This recommendation was based mainly on the studies where purpose was to identify severe anemia with hemoglobin (Hb) <5 grams and moderate anemia with Hb 5–<8 grams [3]. Validity of anemia detection may differ in different settings due to differences in the prevalence of anemia rates, different causes of anemia, and many other factors like different skin pigmentation and so forth that can influence interpretation of palmar pallor. The data about the validity of palmar pallor assessment for detection of anemia from different settings may help improve global understanding about the method of detection. The aim of the present study was thus to study the validity and interobserver agreement of palmar pallor examination to diagnose anemia in children under 5 years of age in India.

## 2. Methodology

In India, every village has Anganwadi centre (AWC), where children of age 6 months–5 years come every morning for 3-4 hours. They are given supplementary food items, immunization, nonformal preschool education, and periodic health check-ups. The study was carried out in a village in northern Haryana, India, with a population of 2500 and two AWCs. A team comprised of a doctor, laboratory technicians, and an attendant visited both of the AWCs on a scheduled day and time. All children aged 6 months–5 years were recruited into the study. Some children who did not report to the AWC were contacted at their homes.

Informed consent was obtained from the parents/guardian to collect capillary blood samples for Hb estimation from their children. A total of 80 children were available in these centres. Trained laboratory technicians obtained blood drop from finger prick by following standard aseptic technique and prepared dried blood sample on filter paper.

In the laboratory, analysis of hemoglobin levels was done by cyanomethemoglobin method using dried blood sample (DBS), within 10 days of receipt of the sample. These dried blood samples on the filter paper were transferred to the test tubes, and the blood sample was extracted using Drabkin's solution. Subsequently, these samples were subjected to laboratory analysis using cyanomethemoglobin method, as used in DLHS4 survey. The laboratory was set up and standardized under DLHS-4 survey and had successfully followed all internal and external quality assurance protocols.

Anemia was defined using different cutoff points to make it compatible with the existing studies and as per the national clinical protocols. Anemia is defined as hemoglobin level <11.0 g/dL and severe anemia as <7.0 g/dL. However, original validation studies had used cutoff of <5 grams for severe anemia and <8 grams for moderate anemia.

Two examiners, a physician and a health worker, previously trained in IMNCI, evaluated clinical signs for these children. The physician was postgraduate in specialty of community medicine and the health worker was graduate working as health worker in village-based health post for more than 10 years. Each examiner was blinded to the other. Pallor was defined as* “some palmar pallor”* if the skin of the child's palm was pale and* “severe palmar pallor”* if the skin of the palm was very pale or so pale that it looked white. Kappa statistics was used to measure the level of agreement between the two examiners. Kappa agreement was also calculated by clubbing the categories of “severe palmar pallor” and “some palmar pallor” as a single category* “pallor*.*”*

Standard measures of a diagnostic test like sensitivity and specificity were also calculated. Single clinical category “pallor” was used to undertake the validation analysis. Validation indices were calculated with this clubbed category using different laboratory cutoffs of Hb levels. Area under ROC curve was also ascertained.

## 3. Results

Out of the 80 children examined, 47 (59%) were males. The age of the children ranged from 6 months to 5 years with mean age of 2.7 years (SD = 1.5). The mean hemoglobin level was 7.0 mg/dL (SD = 1.47) ranging from 4 to 11 mg/dL. Among the children examined, 34 (42.4%) had severe anemia with hemoglobin levels <7 mg/dL, 45 (56.3%) had mild-moderate anemia (7–10.9 mg/dL), whereas only one child (1.3%) had no anemia (≥11 mg/dL).

A health worker could diagnose palmar pallor with sensitivity ranging from 30.8 to 42.8% and specificity from 70 to 89% (excluding extreme value), at different cutoffs of Hb levels. Similar figures for doctor were 40–47% and 60–66%, respectively. Receiver operating curve (ROC) for different cutoffs and both health worker and doctor ranged from 0.51 to 0.75. It was the highest (0.65–0.70) at Hb cutoff of <10 grams (Table 1).

The level of agreement between pallor assessment by a health worker and a physician was found to be 0.482 (95% CI = 0.298–0.666) with weighted Kappa being 0.48. Kappa agreement improved to 0.51 when single category of “pallor” was used to assess the agreement (Table 2).

## 4. Discussion

Our study revealed glaringly very high anemia rate among children 6 months–5 years of age, who were apparently not sick and were attending to the “AWCs.” This may be because of the fact that these centres cater to the poorest section of the society, where anemia rate is expected to be high. High rates of asymptomatic anaemia have been reported in other studies as well, in this part of the world [1, 4, 5].

We found that the sensitivity of palmar pallor was low and specificity was moderate at different cutoffs of Hb levels, used for defining anemia. Variable results have been reported in other studies for classification of anaemia. Montresor et al. [6] and Desai et al. [7] reported a low sensitivity (20–37%) and high specificity (84–91%) of clinical diagnosis. Zucker et al. found that sensitivity for defining severe anemia using cutoff of Hb <5 grams was 60% [8]. In our study sensitivity was 42.8% using the same cutoffs. Luby et al. reported 93% sensitivity for the detection of severe anemia and 66% for moderate anemia [9]. In a meta-analysis, sensitivity of clinical pallor signs ranged from 29.2% to 80.9%. Palmar pallor assessment had the highest pooled sensitivity (80.9%) at hemoglobin < 8 g/dL whereas pooled sensitivity was much lower (39.2%) at hemoglobin threshold level <11 g/dL [3]. We also found similar results; however, differential in sensitivities at different Hb cutoffs was less.

Use of palmar pallor for detection of anaemia in the IMCI guidelines was based on the experience of these African studies, with the understanding that, in resource restraint countries, detection of palmar pallor even at modest level of sensitivity and specificity would be lifesaving for many children. Many authors have argued to improve validity of palmar pallor by combining with other clinical signs [10–15], but these have their own limitations. Therefore, in Indian rural setting, where asymptomatic anemia rate is very high, IMNCI guidelines to detect palmar pallor may still be useful for initiating early treatment for childhood anemia. As per the results of this study, health workers will classify 30% children as “some anemia” or “severe anemia.” All these children will get the therapeutic iron dose correctly, with or without referral. However, 70% children will be labelled as having “no pallor,” who otherwise had some degree of anemia in all but one case. As per IMNCI guidelines these children will also receive dietary counseling and prophylactic iron administration. Second examination by a doctor in such cases is likely to alter the decision from “no pallor” to “pallor” in 13 (16%) cases. But considering comparable sensitivities of health worker and doctor for detection of severe pallor, when laboratory Hb estimation is taken as gold standard, actual contribution of doctor for case detection is likely to be less. It can be argued that in such a setting with very high anemia rate all children may be given therapeutic iron dosage. However, in the absence of any clinical sign of anemia, administration of therapeutic dose of iron may be unethical. Thus we propose that, in settings where it is not possible to do Hb estimations, palmar pallor assessments may be done as per the existing IMNCI protocols. Children with “no pallor” should also be followed up closely and frequently for possible detection of pallor or some other clinical symptom at some later date.

Some limitations of the study are worth noticing. Many factors could have led to variability in validity of the anaemia classification with palmar pallor. Skin colour and palmar pigmentation could vary in different countries. Cleanliness of palms can be a hindering factor for observation. It is likely that sick children who were brought to OPDs or who were examined indoors in other validation studies had clean palms, whereas, in our study, children were examined in their play centres and their hands were likely to be dusty. The effect of extremes of surrounding temperature on palmar blood circulation also cannot be ruled out. A different method of Hb estimation can also account for the variability. It is well documented that HemoCue method as used in some studies gives higher Hb values compared to cyanomethemoglobin method used in our study [16, 17].

Thus, while more research is needed to explore other simple, accurate methods of hemoglobin estimation in high prevalence settings like India, palmar pallor assessment will continue to be useful in early treatment of anemia with the caution that children with “no pallor” should also be followed up closely.

## Figures and Tables

**Table 1:** Validity of doctor and health worker's classification of palmar pallor against different haemoglobin cutoff levels.

	Sensitivity %	Specificity %	Accuracy %	ROC (95% CI)
HB cutoff for anaemia in grams for validation of doctor's classification				
<5	42.8	60.3	58.7	0.51 (0.31–0.72)
<6	43.7	60.9	57.5	0.52 (0.38–0.66)
<7	47.1	65.2	57.5	0.56 (0.45–0.67)
<8	41.1	62.5	47.5	0.52 (0.39–0.63)
<9	40.8	66.7	43.7	0.54 (0.36–0.71)
<10	41	100	42.5	0.70 (0.65–0.76)
HB cutoff for anaemia in grams for validation of health workers classification				
<5	42.8	71.2	68.7	0.57 (0.36–0.77)
<6	31.2	70.3	62.5	0.51 (0.38–0.64)
<7	41.2	78.3	62.5	0.59 (0.49–0.70)
<8	35.7	83.3	50.0	0.59 (0.49–0.69)
<9	32.4	88.9	38.7	0.60 (0.48–0.73)
<10	30.8	100	32.5	0.65 (0.60–0.70)

**Table 2:** Interrater agreement among doctor and health worker to diagnose palmar pallor.

Doctor's classification	Health workers classification			Total
	No pallor	Some pallor	Severe pallor	
No pallor	43 (89.6)	5 (10.4)	0	48
Some pallor	11 (39.3)	16 (57.1)	1 (3.6)	28
Severe pallor	2 (50.0)	1 (25.0)	1 (25.0)	4

Kappa 0.48 (0.09), *P* < 0.001. Agreement 75%, expected 51.7%.

Clubbing some pallor and severe pallor as single category pallor.

Kappa agreement: 0.51 (std. error 0.1), *P* < 0.001. Agreement 77.5%, expected 54%.
